# Physical Activity in Adults With Crohn’s Disease: A Scoping Review

**DOI:** 10.1093/crocol/otac022

**Published:** 2022-06-16

**Authors:** Whitney N Neal, C Danielle Jones, Dorothy Pekmezi, Robert W Motl

**Affiliations:** Department of Health Behavior, University of Alabama at Birmingham, Birmingham, Alabama, USA; Department of Physical Therapy, University of Alabama at Birmingham, Birmingham, Alabama, USA; Department of Health Behavior, University of Alabama at Birmingham, Birmingham, Alabama, USA; Department of Kinesiology and Nutrition, University of Illinois Chicago, Chicago, Illinois, USA

**Keywords:** Crohn’s disease, inflammatory bowel disease, exercise, systematic review

## Abstract

**Background:**

As it becomes increasing clear that managing Crohn’s disease (CD) requires more than medical treatment alone, further research to identify second-line approaches for managing CD and its symptoms such as physical activity (PA) are necessary to address this public health concern.

**Methods:**

We conducted a scoping review of descriptive, cross-sectional, and experimental studies to synthesize evidence regarding PA rates, determinants, health consequences, and interventions specifically in adults with CD. Adhering to the Preferred Items for Systematic Reviews and Meta-Analyses extension for scoping reviews (PRISMA-ScR) guidelines, published literature was searched to identify articles that examined PA or exercise in adults with CD.

**Results:**

Twenty-eight articles met inclusion criteria: 13 included a cross-sectional design, 4 a case–control design, 2 cohort designs, and 9 intervention designs. Adults with CD appear to be similar to somewhat less physically active than the general population, though self-report and objective rates of PA vary widely by geographical location. PA may be associated with the reduced risk of future active disease in adults with CD in clinical remission, as well as improve health-related quality of life, fatigue, cardiorespiratory fitness, and depression. Preliminary findings from interventional studies demonstrate that moderate-intensity PA is feasible, safe, and may have beneficial effects on disease activity.

**Conclusions:**

Overall, the benefits that can be accrued from regular PA are quite diverse; however, a substantially larger body of evidence is needed to provide firmer conclusions on the health benefits of PA that might underlie exercise-related changes in function and disease activity in adults with CD.

## Introduction

Despite the effectiveness of treatments (e.g., corticosteroids, immunosuppressants, biological agents) for inducing long-term remission in adults with Crohn’s disease (CD), secondary disorders such as arthritis, osteoporosis, ocular inflammation, and skin lesions, as well as other extraintestinal symptoms such as fatigue, depression, and anxiety still frequently occur, resulting in a reduced quality of life (QOL).^1^ As it becomes increasing clear that managing CD requires more than medical treatment alone, further research to identify second-line approaches for managing CD and its symptoms are necessary to address this public health concern.

Physical activity (PA) is defined as any bodily movement carried out by contraction of skeletal muscles that results in a substantial increase in energy expenditure over-and-beyond resting levels^2^; exercise, a subset of PA, is planned, structured, repetitive, and purposeful PA intended to improve physical fitness. Emerging evidence indicates that PA and exercise are beneficial in many immune-mediated diseases and conditions (e.g., multiple sclerosis, type 1 diabetes, rheumatoid arthritis), and epidemiological studies have identified an inverse association between PA participation and risk of developing CD.^3–6^ PA guidelines have even been established for adults with general inflammatory bowel disease (IBD) and focus on aerobic activity of 20–60 minutes in duration of 2–5 days per week, complemented by resistance training 2 or more days per week; however, these guidelines were developed before the existence of research investigating the role of PA in IBD patients and are based on the benefits of PA in healthy individuals.^7^

There is an increasing interest by clinicians and researchers regarding the role of exercise and PA in adults with CD. Nevertheless, the existing reviews on PA and its benefits in IBD oftentimes group CD and ulcerative colitis (UC) together. CD and UC are both chronic and relapsing diseases involving inflammation in the bowels, but CD and UC differ in important ways necessitating a focal review on PA in CD. For example, CD is transmural and occurs along any part of the gastrointestinal tract, whereas UC occurs in the colon and mucosa and superficial submuscosa.^8,9^ The symptoms of CD are therefore more heterogeneous than those of UC, with extraintestinal symptoms (e.g., aching, painful joints, osteoporosis, skin lesions, fatigue, depression) occurring more frequently in CD than UC.^8^ Due to its transmural nature, the development of strictures or fistulae (generally to the bowel or bladder) are more common in CD than UC.^8,9^ Aerobic exercise capacity measured by cardiopulmonary exercise testing appears to be reduced in adults with IBD; however, after adjusting for age and sex, CD patients have lower exercise capacity compared to both those with UC and reference values.^10^ Such differences support the unique consideration of PA in CD, rather than reliance upon reviews of PA that consider CD and UC together.

Accordingly, we conducted a scoping review of descriptive, cross-sectional, and experimental studies to summarize existing evidence regarding PA rates, determinants, health consequences, and interventions specifically in adults with CD. The goal was to provide an overview of PA patterns and potential barriers to PA, the role of PA in CD disease course, health outcomes of PA in CD, and the effects of structured PA interventions on clinical parameters of CD. The current scoping review is a critical first step in establishing a research agenda for PA promotion, creating tailored PA interventions, and developing evidence-based PA guidelines for adults with CD.

## Methods

### Scoping Review

Scoping reviews provide a broad overview of the available research and compile the main sources and types of evidence available, in contrast to systematic reviews that produce a summary of the effectiveness of a particular intervention based on a precise set of outcomes.^11^ This is important as the drive toward evidence-based practice has gathered pace, and new approaches toward evidence synthesis, such as scoping reviews, have emerged in recent years for informing clinical research and practice in chronic diseases and conditions.^12^ This scoping review was structured a priori based on the framework created by Arksey and O’Malley^13^ and further refined based on the Joanna Briggs Institute,^12^ and followed a 5-step process: (1) identifying the questions, (2) identifying relevant studies, (3) study selection, (4) charting the data, and (5) collating, summarizing, and reporting results. This review further adhered to the Preferred Items for Systematic Reviews and Meta-Analyses extension for scoping reviews (PRISMA-ScR) guidelines.^14^

### Search Strategy and Study Selection

**Figure 1.**
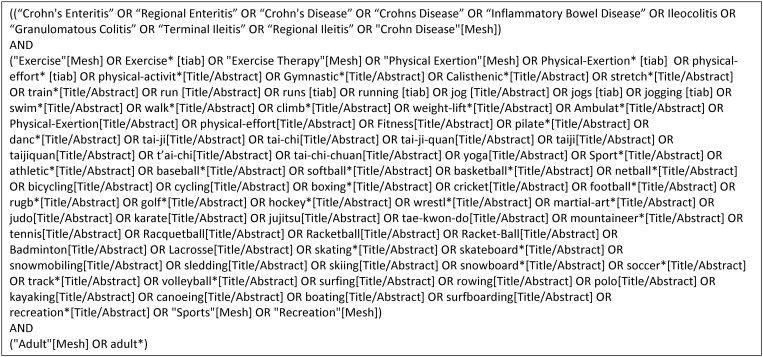
Screenshot of the PubMed literature search strategy.

Two primary searches were conducted to retrieve high quality, peer-reviewed publications that examined PA and CD. The preliminary search was performed in April 2021 and a follow-up search was undertaken in August 2021. The preliminary search focused on PubMed, Embase, and Google Scholar databases with the terms “Crohn’s disease,” “Crohn’s,” “exercise,” and “physical activity.” These databases were selected to provide a broad range of results related to the topic of interest. The follow-up search used the same databases but, with the assistance of a librarian, subject headings were combined into search strings for each of the 3 electronic databases. A typical search string included the subject headings “Crohn’s disease,” “physical activity” and “exercise,” and “adults” (Figure 1). The Boolean operator OR was used to maximize the searches and the operator AND was used to combine terms. To maximize the number of publications retrieved, papers found in reference lists of included publications were also included in the review.

Studies were included that (1) targeted adults (≥18 years) diagnosed with CD, (2) included descriptive, correlational, or experimental clinical trial designs that focused on PA or exercise, and (3) published in English from the period of inception to August 30, 2021. The exclusion criteria were: (1) literature reviews and (2) non-peer-reviewed publications (i.e., commentary papers, dissertations, and conference presentations). As there are a limited number of publications examining PA and CD, no studies were excluded from the review based on publication date or study design; however, rigor of design was considered when reviewing/interpreting study findings.

### Data Charting and Collation

The eligible studies were coded independently by the first and second authors using Covidence and organized into tables. Data extracted included participant demographic and clinical characteristics (age, sex, CD disease activity); intervention characteristics (type, duration, setting, country of origin, and mode of delivery); participant flow (frequency count for the people screened, enrolled, and completed the study); and outcomes relevant to the review questions. First, the research design of each article was grouped into one of the following categories: (1) cross-sectional, (2) cohort, (3) case–control, or (4) intervention. Second, primary and secondary outcomes were coded into one of the following categories related to the aims of this review: (1) PA rates, (2) PA correlates or predictors, (3) outcomes or consequences of PA, or (4) PA interventions. Finally, interventions were coded by the PA category focus of the intervention (e.g., aerobic exercise, resistance training, or a combination).

Study outcomes were grouped into respective categories and reported as primarily significant or nonsignificant. Authors deemed outcomes as significant based on 2 criteria: (1) a significant outcome related to the study’s primary objective or (2) at least half of the reported outcomes within the outcome category were significant. The sample size value was calculated by summing all adults with CD recruited in each study group. In some cases, a single trial was associated with multiple publications that examined different outcomes; these publications were recorded as a single trial when recording frequency counts of participant characteristics.

## Results

**Figure 2.**
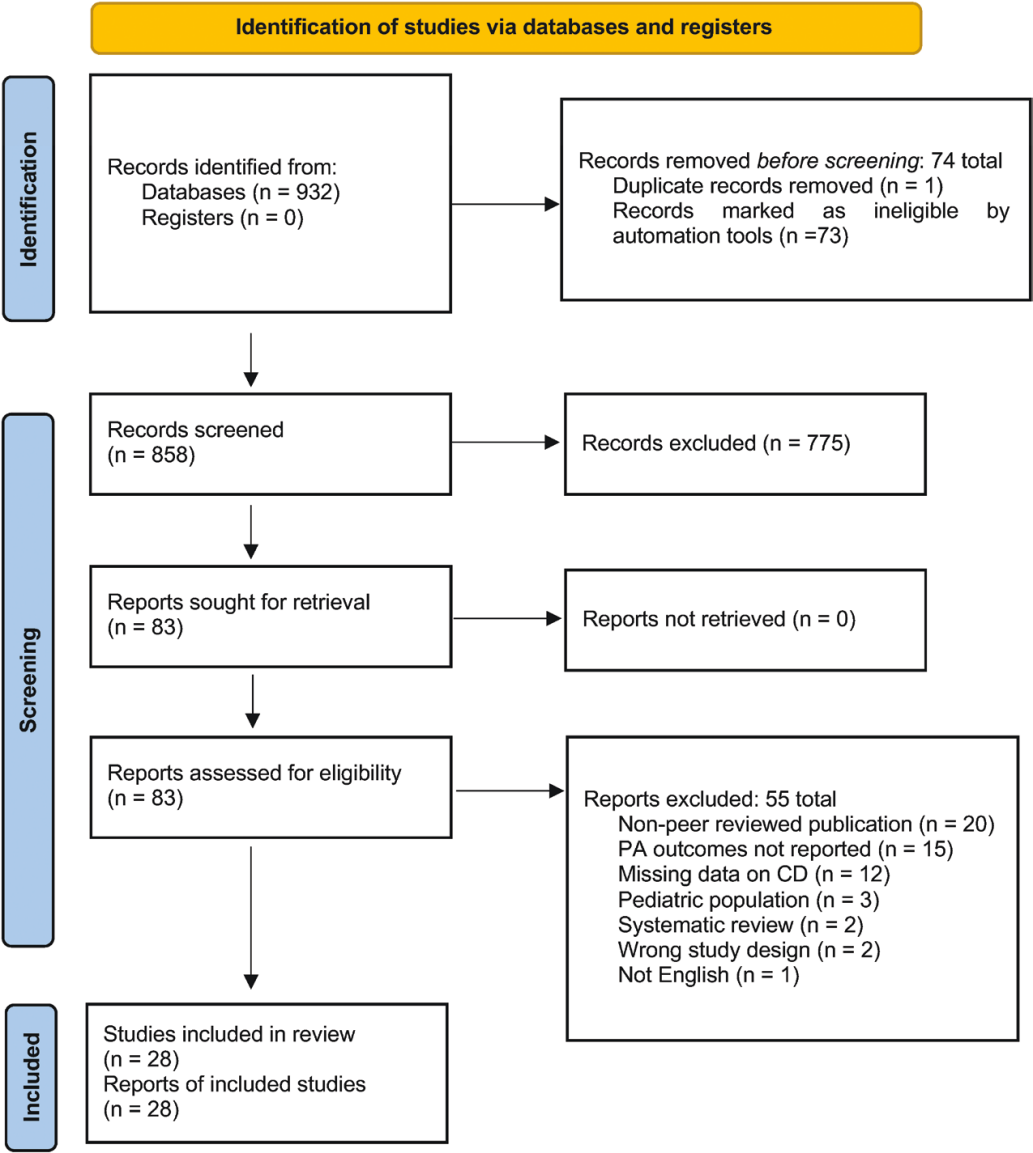
PRISMA 2020 flow diagram of literature search and study selection.

Results of the article selection process are provided in the PRISMA flowchart in Figure 2. Of the 932 records identified, 74 duplicate records were removed, leaving 858 records. Of these, 775 records were excluded based on title and abstract screening. The remaining 83 studies were screened for eligibility at the full-text level. During full-text reviews, 56 articles were excluded; however, after further discussion between the first and second authors, 1 article that was originally excluded was included after all due to relevance. This resulted in 28 articles that met inclusion criteria for the analysis (Figure 2).

### Study Characteristics

Twenty-eight papers were included in this review; 13 included a cross-sectional design, 4 a case–control design, 2 cohort designs, and 9 intervention designs. Geographical locations of the research studies included the United Kingdom,^15–21^ Canada,^22–24^ Brazil,^25,26^ Korea,^27,28^ the Netherlands,^29,30^ and the United States,^31,32^ with 1 study each from Australia,^33^ Croatia,^34^ Denmark,^35^ France,^36^ Germany,^37^ Malta,^38^ New Zealand,^39^ Italy,^40^ Jordan,^41^ and Poland.^42^ Included studies were conducted between 1987 and 2021, with most (*n* = 16) of these studies published within the last 5 years. Of note, 2 of the 28 selected papers were secondary data analyses (i.e., reporting on different PA outcomes from an original intervention),^16,19^ resulting in 26 research studies in this scoping review.

The current review presents data from a total of 3691 adults with CD. The diagnosis of CD was confirmed via self-report (*n* = 4)^22,30–32^ or clinical data (*n* = 21)^15–21,23,25–29,33–42^; it was unclear how CD was confirmed in 1 study.^24^ Sample sizes varied considerably, ranging between 6^40^ and 1308^31^ persons with CD. The mean (standard deviation) age of participants with CD was 39.8 (±4.86) years and approximately 61% of participants were female.

### PA Rates

Eleven studies provided data on rates of PA in CD. Ten of the studies measured PA using subjective measures (modified Baecke questionnaire,^25,36^ Godin Leisure-Time Physical Activity Questionnaire [GLTEQ],^27,28^ International Physical Activity Questionnaire [IPAQ],^18,32,39^ modified Physical Activity Monitor,^22^ 7-day Physical Activity Recall [PAR] questionnaire,^41^ tabulated participant PA levels based on semistructured interviews^35^), and 3 reported device-measured PA^25,33,36^ (Table 1). When compared with the general population, adults with CD generally had similar or lower rates of PA.^22,25,33,36,41^ Adults with CD were markedly less active (58.0% inactive, 24.1% moderately active, 17.9% active vs 50.8% inactive, 25.2% moderately active, 24.1% active, and 1818.8 ± 887.2 vs 2479.4 ± 296.8 METS week^−1^; *P* = .033)^22,41^ than matched healthy controls. Device-based measures of PA, namely accelerometers, indicated that participants with CD demonstrated significantly fewer total accelerometer counts compared with healthy controls (1.32 × 10^6^ vs 1.95 × 10^6^; *P* < .01).^33^ Two studies that utilized both self-report and device measures of PA found no differences in PA between the CD and control group^25,36^; however, the CD group spent more time lying down based on the accelerometer data (116.3 ± 107.3 vs 63.7 ± 55.7 min d^−1^, *P* = .046) in 1 study.^25^

**Table 1.** Overview of observational studies examining rates of physical activity in Crohn’s disease.

Reference, year	Country	PA measure/device	Number of participants	Results
Chae, 2016	Korea	GLTEQ	62 CD	Mean duration of weekly PA = 96.3 min
Crumbock et al., 2009	United States	IPAQ	17 CD	52.9% high PA, 23.5% moderate PA, 23.5% low PA
Fagan, 2021	New Zealand	IPAQ-SF	46 CD	69% of CD patients met PA guidelines
Kim, 2021	Korea	GLTEQ	59 CD	Mean duration of weekly PA = 96.3 min
Tew, 2016	United Kingdom	IPAQ-SF	446 CD	21.7% of mildly active CD physically inactive vs 62.1% of severely active CD physically inactive
Cabalzar, 2019	Brazil	Modified Baecke, accelerometer	26 CD Comparison: functional dyspepsia patients	CD ppts spent more time lying down (116.3 ± 107.3 min/d vs 63.7 ± 55.7 min/d, *P* = .046)
Mack, 2010	Canada	Modified Physical Activity Monitor	479 CDControls: HCs	58.0%, 24.1%, 17.9% of CD patients inactive, moderate LTPA, active LTPA, respectively, vs 50.8%, 25.2%, 24.1% of healthy controls inactive, moderate LTPA, active LTPA; *P* < .05
Qalqili, 2021	Jordan	PAR	85 CD Controls: 150 HCs	Adults with CD were significantly less active than those without CD (1818.8 ± 887.2 vs 2479.4 ± 296.8 METS wk^−1^; *P* = .033)
Sorensen, 1987	Denmark	Interviews	106 CD Comparison: 75 previously hospitalized patients	Similar levels of PA in both groups (CD group: 26 high PA, 73 moderate PA, 7 low PA vs HC group: 22 high PA, 48 moderate PA, 5 low PA)
Van Langenberg, 2015	Australia	Accelerometer	48 CD Controls: 30 HCs	CD ppts were significantly less active than healthy controls (1.32 × 10^6^ (3.45 × 10^4^–4.13 × 10^6^) vs 1.95 × 10^6^ (1.10 × 10^6^–3.70 × 10^6^), *P* < .01)
Wiroth et al., 2005	France	Modified Baecke, accelerometer	41 CD Controls: 25 HCs	Similar 7-day accelerometer counts and total questionnaire index; lower sport (2.41 ± 1.10 vs 2.66 ± 0.74, *P* < .05) and higher work (2.94 ± 0.80 vs 2.51 ± 0.44) index in CD vs HC group

Abbreviations: CD, Crohn’s disease; GLTEQ, Godin Leisure-Time Exercise Questionnaire; HC, healthy control; IPAQ, International Physical Activity Questionnaire; IPAQ-SF, International Physical Activity Questionnaire-Short Form; LTPA, leisure-time physical activity; MET, metabolic equivalent; PA, physical activity; PAR, 7-day Physical Activity Recall questionnaire.

There was some variation in rates and types of PA by country. For example, a Denmark study that used a control group consisting of patients previously hospitalized for acute disease (i.e., tonsillitis, pneumonia) reported similar PA rates between groups.^35^ Two studies reported that Korean adults with CD exercised on average 96.3 min week^−1,27,28^ well below recommended guidelines of at least 150 minutes of moderate-intensity aerobic PA/week.^43^ A French case–control study comparing habitual PA rates between CD patients and healthy controls reported similar overall levels of PA; however, further analysis indicated lower sport and higher work index scores when compared to healthy controls. In the United States, 52.9% of adults with CD reported being engaged in high levels of PA, 23.5% were moderately PA, and 23.5% participated in low levels of PA.^32^ By comparison, a New Zealand survey assessing PA habits reported that 70% of adults with CD were currently meeting PA guidelines,^43^ with 24% and 46% of participants reporting high and moderate levels of PA, respectively.^39^ This is similar to an earlier UK study categorizing 21.7% of participants with mild CD (defined as a 3-item Patient-Reported Outcome [PRO3] score between 13 and 21) as physically inactive, although 62.5% of participants with severely active CD (PRO3 ≥52) were classified as inactive,^18^ suggesting a link between PA rates and disease activity.

### Correlates and Predictors of PA

Table 2 summarizes variables associated with PA levels in adults with CD. A younger age at diagnosis^38^ and high prediagnosis PA levels^31,38^ were each independently associated with lower levels of PA postdiagnosis. Longer disease duration, presence of active disease (as per serum C-reactive protein [CRP] >3 mg L^−1^), and low vitamin D3 (<50 nmol L^−1^) were further associated with lower PA.^33^ Nonetheless, the correlation between PA and self-reported disease activity in CD is unclear, as 3 cross-sectional studies reported correlations between clinical disease activity^18,30,31^ with PA (all *P* = .01), whereas 2 other studies did not.^32,33^

**Table 2.** Overview of observational studies examining variables associated with physical activity in Crohn’s disease.

Reference, year	Study location, type, methodology	Physical activity measure/device	Results
van Langenberg et al., 2015	Australia. Cross-sectional, CD patients’ sleep quality and PA compared to matched HCs	Accelerometer	No correlation between disease activity and PA (*P* = .24); longer duration since CD diagnosis, presence of inflammation, and low vitamin D3 were all independently associated with lower PA.
Crumbock et al., 2009	United States. Cross-sectional study via postal questionnaire	GLTEQ	No correlation between disease activity and PA
Gatt et al., 2019	Malta. Cross-sectional study assessing pre- and postdiagnosis PA levels.	GLTEQ	Younger age at diagnosis and a high premorbid GLTEQ score each independently predicted change in GLTEQ score after diagnosis.
Jones et al., 2015	United States. Cohort study, examined disease activity of physically active and inactive patients with CD	GLTEQ	Correlation between PA and active disease (*P* = .01)
Tew et al., 2016	United Kingdom. Cross-sectional study. Patients were nonclinical population recruited through online advertisement	IPAQ-SF	Correlation between disease activity (*P* < .01), depression (*P* < .01), anxiety (*P* < .05), fatigue (*P* < .05), exercise benefits score (*P* < .01), and exercise barriers score (*P* < .01)
Lamers et al., 2021	Netherlands. Cross-sectional study. PA and disease activity assessed via online survey.	SQUASH	Inverse association between PA and disease activity (*P* = .013)

Abbreviations: BMD, bone mineral density; CD, Crohn’s disease; GLTEQ, Godin Leisure-Time Exercise Questionnaire; HC, healthy control; IPAQ-SF, International Physical Activity Questionnaire-Short Form; PA, physical activity; SQUASH, Short Questionnaire to Assess Health-enhancing Physical Activity; UC, ulcerative colitis.

Finally, a cross-sectional study identified significant correlations between PA and depression, anxiety, fatigue, exercise benefits, and exercise barriers.^18^ Three hundred and sixty-two participants (81%) reported that CD limited participation in PA/exercise. The most commons reasons were abdominal or joint pain (81%), fatigue or tiredness (85%), disease flare-up (65%), and increased toilet urgency (70%). Other barriers to PA included nausea (42%), muscle weakness (39%), and lack of toilet access (37%).^18^

### Health Consequences of PA

Adults with CD who were PA reported better physical and mental health and lower disease activity (Table 3). One cohort study examined associations between PA and subsequent risk of active disease relapse in 1308 CD patients in remission. Higher PA (measured by the GLTEQ) was associated with a 32% reduction in risk of active disease after 6 months (RR = 0.72, 95% CI: 0.55–0.94, *P* = .02).^31^ Conversely, in a cohort study assessing the relationship between disease activity and PA, adults who achieved remission had a significant increase in steps walked per day (*P* = .006) and reduced inactive time (*P* = .033).^26^ Inactive adults with CD reported higher disease activity,^18,34^ whereas those with higher levels of PA experienced better QOL and health-related quality of life (HRQOL),^32,39^ as well as lower rates of depression and fatigue.^18,39^ When evaluating the effects of moderate-intensity aerobic PA on inflammatory response and disease activity, no statistically significant changes in inflammatory markers nor disease activity after 1-hour of cycling on a leg ergometer were detected.^40^

**Table 3.** Health outcomes of physical activity in Crohn’s disease.

Reference, year	Study location, type, methodology	Physical activity measure/device	Outcomes of physical activity
**Patient-reported outcomes**			
Crumbock et al., 2009	United States. Cross-sectional, information obtained via online survey	IBDQ	↑ QOL (*P* = .022)
Fagan et al., 2021	New Zealand. Cross-sectional study investigating current PA levels	IBDQ	↑ QOL (*P* = .012) and ↓ fatigue (IBD-F, *P* = .043; MFI-physical, *P* = .012)
Holik et al., 2019	Croatia. Cross-sectional, therapy-free CD patients	Questionnaire	↓ disease activity (*P* < .001)
Jones et al., 2015	United States. Cohort study, examined disease activity of physically active and inactive patients with CD	GLTEQ	↓ risk of active disease (RR = 0.72, 95% CI: 0.55–0.94, *P* = .02)
Tew et al., 2016	United Kingdom. Cross-sectional study. Patients were nonclinical population recruited through online advertisement	HADS	↓ disease activity (*P* = .038), ↓ rates of depression (*P* = .002) and fewer perceived PA barriers (*P *= 0.008)
**Clinical outcomes**			
D’Inca et al., 1999	Italy. Cross-sectional, GI parameters measured after 1 h of PA at 60% VO_2_ max	GXT	No Δ in GI parameters or disease activity immediately or after 6 months
Lucca et al., 2020	Brazil. Cohort study, followed CD patients 6 months post-infliximab induction therapy	Accelerometer	↑ steps (*P* = .006), ↓ inactive time (*P* = .033) in those who achieved remission
Robinson et al., 1998	United Kingdom. Cross-sectional study	ADNFS	PA not significantly associated with low BMD
Rychter et al., 2021	Poland. Cross-sectional study	Questionnaire	CD patients with osteopenia or osteoporosis showed less PA when compared to CD/UC patients with normal BMD (*P* = .0335)

Abbreviations: ADNFS, Allied Dunbar National Fitness Survey; BMD, bone mineral density; CD, Crohn’s disease; GI, gastrointestinal; GLTEQ, Godin Leisure-Time Exercise Questionnaire; GXT, Graded exercise test; HADS, Hospital Anxiety and Depression Scale; IBD-F, Inflammatory Bowel Disease Fatigue questionnaire; IBDQ, Inflammatory Bowel Disease Questionnaire; MFI, Modified Fatigue Index; PA, physical activity; QOL, quality of life; UC, ulcerative colitis.

Mixed findings arose from 2 cross-sectional studies that directly examined PA levels and bone health in CD. The first assessed PA using the Allied Dunbar National Fitness Survey and, although low bone mineral density (BMD) was present in 40% of participants, the relationship between PA and BMD was not statistically significant.^20^ Another recent study reported significantly lower PA levels in CD patients with osteoporosis or osteopenia when compared to CD and UC patients with a normal BMD (*P* = .0335); however, this study utilized a questionnaire with unknown psychometric properties when assessing PA.^42^

### PA Interventions

There were 7 different PA interventions, focused on exercise training, that were examined across 9 of the 28 papers in this review,^15–17,19,21,23,24,29,37^ including 5 randomized controlled trials (RCTs),^15,17,21,23,37^ 1 non-RCT,^29^ and 1 single group, pre- to post-test design.^24^ Exercise modalities represented 1 of 3 categories: aerobic (*n* = 4), strength (*n* = 2), or combined aerobic and strength (*n* = 1). The exercise interventions ranged in durations from 4 days to 12 months, with most interventions lasting 12 weeks (*n* = 4).^15,23,24,37^ A comprehensive summary of the interventions is presented in Table 4.

**Table 4.** Overview of interventional studies examining the impact of exercise on Crohn’s disease.

Reference, year	Country	Design	Sample size	Intervention	Outcomes	Results
Jones et al., 2020	United Kingdom	RCT	47	60 min combined impact and resistance training 3×/wk for 6 m	BMD, muscle function, QOL	↑ BMD, muscle function, HRQOL (*P* = .001); ↓ fatigue severity in PA group
Loudon et al., 1999	Canada	Single group	12	12 wk group walking program 3×/wk	Disease activity, QOL, cardiorespiratory fitness	↓ stress (*P* < .001), disease activity (*P* = .02); ↑ QOL (*P* = .01), VO_2_ max (*P* < .01)
Ng et al., 2007	Canada	RCT	32	30 min low-intensity walking 3×/wk at 60% HR max for 12 wk	Disease activity, QOL	↑ QOL (*P* < .05) with no Δ in disease activity, ↓ symptoms (*P* < .01)
Robinson et al., 1998	United Kingdom	RCT	117	12 m home-based low-impact resistance training program completed 2×/wk	BMD	↑ BMD at hip and lumbar spine in PA group, not significant when analyzed on intention-to-treat basis. ↑ BMD at greater trochanter in fully compliant participants (≥10 sessions/month). ↑ BMD at hip & spine positively associated with # completed PA sessions + # reps for each exercise
Watters et al., 2010	United Kingdom	RCT	Same as Robinson et al.	Same as Robinson et al.	Wellbeing	↑ adherence correlated with ↑ illness acceptance and life satisfaction; baseline illness acceptance predicted PA uptake
Seeger et al., 2020	Germany	RCT	45	30 min home-based aerobic or bodyweight activity 3×/wk for 12wks	Disease activity, inflammatory parameters, PA, QOL, strength	↑ in maximal and average strength in both PA groups (*P* = .04); ↑ emotional function in endurance training group (*P* = .03); ↑ PA in both PA groups at 6 months (*P* = .01); ↑ QOL
Tew et al., 2019	United Kingdom	RCT	36	30 min cycling at 90% (HIIT) and 35% (MICT) peak power 3×/wk for 12 wk	Cardiorespiratory fitness	↑ VO_2_ in PA groups, higher in HIIT group; no Δ in behavioral parameters
Bottoms et al., 2019	United Kingdom	RCT	Same as Tew et al.	Same as Tew et al.	Wellbeing	↑ feeling state and enjoyment similar in both groups
Lamers et al., 2020	Netherlands	Non-RCT	16	30, 40, or 50 km of walking at self-selected pace on 4 consecutive exercise days	Disease activity	Similar HBI scores at baseline (*P* = .82) and postintervention (*P* = .10) between groups; no Δ in control group (*P* = .50); ↑ HBI scores over time in PA group (*P* = .024), significant difference between groups (*P* = .046)

Abbreviations: BMD, bone mineral density; HBI, Harvey–Bradshaw Index; HIIT, high-intensity interval training; HR, heart rate; HRQOL, health-related quality of life; MICT, moderate-intensity continuous training; PA, physical activity; QOL, quality of life; RCT, randomized controlled trial.

The aerobic exercise interventions consisted of either walking^23,24,29^ or cycling on a leg ergometer.^15,16^ All but one of the interventions prescribed 12 weeks of aerobic exercise 3 times a week,^15,23,24^ and 1 was a group-based PA intervention.^24^ Two of the walking RCTs observed significant improvements in QOL^23,24^; the group walking program further reported increases in cardiorespiratory fitness (VO_2_ max) and reductions in disease activity and stress.^24^ The supervised high-intensity interval training (HIIT) and moderate-intensity continuous training (MICT) program (the EXACT study) completed on a leg ergometer^15,16^ achieved significant improvements in VO_2_ max in both the HIIT and MICT groups when compared to the control group; the increase in VO_2_ max was larger in the HIIT group than MICT. An ancillary study compared affective (i.e., pleasure and displeasure) and enjoyment responses to HIIT and MICT using the 1-item Feeling Scale (FS) and Physical Activity Enjoyment Scale (PACES) and identified similarly high feeling state and enjoyment responses in both groups.^16^

On the other hand, the third aerobic exercise intervention assessed the effects of repeated bouts of walking on inflammatory markers (i.e., cytokines and fecal calprotectin) and clinical disease activity (Harvey–Bradshaw Index [HBI]) in adults with CD utilizing a nonrandomized design.^29^ Results indicated similar disease activity between CD walkers and CD nonwalkers at baseline and postintervention. HBI scores remained stable over time in nonwalkers; however, disease activity increased significantly over time in CD walkers (*P* = .024) and therefore became significantly different between CD walkers and CD nonwalkers (*P* = .046), indicating that clinical disease activity worsened in the PA group. Nonetheless, fecal calprotectin was not affected by PA and changes in cytokine concentrations were similar for CD walkers and non-CD walkers, suggesting that this type of PA does not lead to disease exacerbation.

Both strength training RCTs evaluated the impact of a home-based resistance training program on BMD in adults with CD. The first strength training intervention consisted of a 12-month home-based low-impact resistance training program aimed at increasing BMD in adults with quiescent or mildly active CD.^19,21^ Those randomized to the PA group completed floor-based low-impact exercises focused on the hip and lumbar region (i.e., quadriceps, hamstrings, gluteal, erector spinae, and muscles of the interior wall) twice a week for 12 months. Results indicated an increase in BMD at the hip and lumbar spine in the PA group when compared to controls; however, retention was problematic (<63% of participants completed the intervention) and no significant difference was detected when analyzed on an intention-to-treat basis.^21^ Nonetheless, PA group participants experienced increases in illness acceptance and life satisfaction postintervention^19^; baseline illness acceptance further predicted exercise uptake and was associated with the reported number of completed exercise sessions at 12 months.^21^ The PROgressive resistance Training Exercise and Crohn’s disease Trial (PROTECT) examined the effects of a 6-month home-based resistance training program on BMD and muscle function in adults with CD.^17^ The program consisted of impact (i.e., skipping rope, several multidirectional jumps) and high-effort resistance exercises targeting the major muscle groups of the upper-body, lower-body, and core (e.g., squat, lunge, reverse fly, lateral raise, bridge, etc.) performed 3 times per week for 60 minutes. Participants received TheraBands, a jump rope, and an exercise booklet containing instructions for performing the exercises and tables for self-monitoring. Results indicated greater improvements in HRQOL, fatigue, BMD, and all muscle function outcomes (i.e., grip strength, lower- and upper-body muscular endurance, isokinetic strength of knee extensors and elbow flexors) at 6 months in the exercise group compared to the control group.

One RCT evaluated the effects of a 12-week home-based moderate aerobic exercise and moderate resistance training program on disease activity in physically inactive (PA less than 2 times per week for less than 60 minutes) adults with CD.^37^ Participants were randomly assigned to either a moderate endurance exercise training, moderate muscle training, or control group. Both groups exercised independently 3 times per week; participants in the moderate endurance training group completed their preferred form of aerobic exercise (i.e., jogging, cycling, walking), and the muscle training group performed bodyweight exercises. Both PA groups experienced significant improvements in muscle function (i.e., grip strength, quadriceps strength; all *P* < .04) and QOL and reported higher levels of PA at follow-up after 6 months (*P* = .01). The endurance training group further experienced greater improvements in emotional function when compared to the muscle training and control groups (*P* = .03). It is, however, worth noting that the dropout rate in the muscle training group was significantly lower compared to the endurance group (*P* = .04) with 86.7% of muscle training group participants and only 52.9% of endurance group participants completing the study.^37^

## Discussion

This scoping review summarized the current state-of-the-science regarding PA rates, correlates, outcomes, and interventions that have been conducted in adults with CD. Overall, despite reduced rates of PA in adults with CD, the findings highlighted the positive effects of PA on HRQOL; the improvement of physical and mental health; the reduced risk of future active disease; and the reduction of CD symptoms. Below, we discuss several knowledge gaps in the included studies and potential solutions to (1) better understand the daily PA patterns and determinants of PA in adults with CD, and (2) gain a better understanding of the CD-related health outcomes that can be influenced by regular PA.

### Rates and Determinants of PA in CD

One goal of this review involved compiling knowledge on the rates of PA in CD. Results indicate that rates of PA in adults with CD are similar to or somewhat lower than the general population, though rates vary widely by geographical location. Adults with CD further reported engaging in less PA than those with UC.^22,27,28,30,41^ Importantly, there was considerable heterogeneity in how the studies quantified PA (i.e., self-report or objective measurement), with 9 studies using 6 different self-report measures of PA.^18,22,25,27,28,32,35,39,41^ There was further an inconsistent pattern of results pertaining to PA rates of persons with CD when compared to the general population, with 2 studies incorrectly reporting the use of healthy control comparison groups; instead, the comparison groups consisted of patients with functional dyspepsia^25^ and those recently hospitalized for acute disease^35^; not surprisingly, both studies reported similar rates of PA in the CD and comparison groups. To the best of our knowledge, only 2 US studies have examined PA in adults with CD, and only 1 reported rates of PA.^31,32^ Collectively, such a paucity of consistent data makes it difficult to draw definitive conclusions on PA levels of adults with CD worldwide. Future studies are necessary to better comprehend the sources of unexplained variance in PA levels as this represents a meaningful step in health promotion efforts in this population.

Another goal involved a review of correlates of PA levels in CD. Disease activity is 1 factor that may explain the wide variability of PA patterns across studies included in this scoping review. Several clinical indices have been used in studies to assess disease activity in CD, including the HBI and CD Activity Index (CDAI) to measure subjective clinical symptoms, and more objective measures such as CRP and fecal calprotectin^44–46^ as biomarkers of disease activity. The CDAI has evolved as the gold standard for clinical trials^47^; however, the accuracy of the CDAI to detect biomarker remission is relatively low and rarely used in clinical practice.^48^ In this scoping review, 1 study identified an association between accelerometer-measured PA and presence of inflammation, but not disease activity (CDAI).^33^ Participants in the 4-day walking intervention reported significant increases in disease activity (HBI) postintervention, but researchers observed no changes in inflammatory markers.^29^ In a large cross-sectional study conducted in the United Kingdom, higher disease activity (PRO3) was associated with lower PA, with 41% more patients with severely active disease categorized as inactive compared to adults with mildly active CD.^18^ Taken together, these data suggest that CD clinical disease activity is virtually independent of the severity of biological markers of disease activity. Nevertheless, several factors can influence PA participation during active disease including bowel urgency, pain, fatigue, and disease flare-up.^18,49^ Conversely, those with mild or inactive form of disease activity may engage in higher levels of PA thus improving health outcomes. Overall, it seems like PA is negatively associated with biomarkers of disease activity, suggesting it can be considered feasible and safe for people with CD; however, future trials should utilize both self-report and objective measurements of disease activity to examine the relationship between PA and disease severity.

### Health Benefits of PA in CD

This review examined the health benefits of PA in CD. Adults with CD who were PA reported better HRQOL, fatigue, and depression. The HRQOL of adults with CD is particularly important given the young age at onset, impact on productivity, and recurrent nature of the disease. HRQOL refers to the functional impact of an illness on physical function, emotional/social function, ability to work productively, and absence of disease-related symptoms.^50^ Although disease activity is an important determinant of HRQOL, even asymptomatic adults with CD report lower HRQOL, suggesting a role of other determinants. Two cross-sectional studies reported that physically active adults with CD reported higher HRQOL,^32,39^ and all 4 interventions that examined QOL/HRQOL reported significant improvements in the PA group, suggesting a beneficial effect of regular PA on HRQOL in people with CD.^17,23,24,37^ However, more RCTs are needed to determine its true effectiveness.

Adults with CD have higher rates of osteoporosis and osteopenia than the general population, with 1 cross-sectional study reporting low BMD in 40% of participants^20^ and reports that up to 80% of patients with CD suffer some degree of bone loss.^51^ Research further suggests that adults with CD are at a small but significantly increased risk of fracture compared with healthy controls and adults with UC.^51^ Despite the well-documented benefits of PA on bone parameters in healthy populations, there has been very little investigation into the benefits of PA for the prevention and treatment of osteoporosis and osteopenia in adults with CD. The etiologies behind the development of bone loss in CD are multifaceted, likely due to issues such as chronic inflammation, weight loss, genetic susceptibility, malabsorption, and reduced PA.^51,52^ Few treatment options exist for CD patients presenting with significant bone loss; therefore, the role of PA, particularly weight-bearing exercises, in the prevention and treatment of osteoporosis and osteopenia secondary to CD may be important as an adjunct to traditional therapy such as calcium supplementation. The 2 studies included in this scoping review that investigated the relationship between PA and BMD in CD reported conflicting results,^20,42^ but only 1 study utilized a validated questionnaire to assess PA.^20^ Future research should examine the impact of PA for the prevention and treatment of bone loss in adults with CD using validated measures of PA.

### PA Interventions for Adults With CD

We lastly reviewed interventions involving PA in CD. Interventions targeted relevant health conditions associated with CD, mostly examining QOL and disease activity as outcomes. Study findings demonstrate that moderate-intensity PA is feasible, safe, and may have beneficial effects on the disease course, QOL, BMD, muscle function, and cardiorespiratory fitness of adults with CD. Only 1 intervention reported slightly negative effects immediately postintervention (increased disease activity in the PA group), though they did not impact PA participation.^29^ Nearly all interventions prescribed 3 sessions per week of PA^15–17,23,24,37^; participant interviews indicated mixed views about this frequency, with some stating that 3 sessions per week is difficult to adhere to, whereas other participants felt that this frequency was achievable and necessary for accruing health benefits. Walking, cycling, and resistance training were the most common modes of PA prescribed. Interestingly, of the 27 participants in the EXACT study who expressed a preference for a particular group before allocation, most (74%) preferred HIIT.^15^ Importantly, although 1 participant from the HIIT group in this study experienced a relapse, the relapse was unrelated to the program, suggesting that high-intensity PA is also safe in adults with CD and can result in improvements in aerobic capacity.^15^

Preliminary evidence suggests that aerobic exercise capacity (i.e., anaerobic threshold and peak exertional oxygen consumption) is reduced in adults with CD,^10^ with 1 study reporting that in postoperative patients with CD, aerobic capacity appears to be reduced in proportion to the extent of past bowel resection when compared to matched healthy controls.^53^ Skeletal muscle mass and strength are further reduced^36,52^ and local muscle fatigue is increased^54^ in CD patients in remission.^52,54^ Some data suggests that lower levels of moderate-to-vigorous intensity PA may have deleterious effects on muscle performance in adults with CD, irrespective of global habitual PA levels.^36^ This topic is of importance because reduced cardiorespiratory fitness and muscle weakness are both significant health risks that may contribute to a poor HRQOL. Three RCTs observed significant improvements in aerobic capacity and muscle strength, indicating a positive relationship between PA and these physical parameters.^23,24,37^

Surprisingly, basic guidelines specifically for CD patients promote the benefits of PA for improving overall health, recommending 20–30 minutes of low-intensity walking at 60% of maximal heart rate 3 days every week and resistance exercises focused on the major muscle groups of the trunk and legs at 50% of one-maximum repetition (1-RM) at least twice a week; however, these recommendations are based on preliminary evidence of the impact of PA on CD and have yet to be integrated into the accepted literature.^55^ Future research should examine the feasibility and outcomes of delivering these guidelines in adults with CD.

### Limitations

There are several limitations of this scoping review. Electronic database searches were conducted with extensive, a priori search criteria, and this may have biased the resultant studies that were included for review. Unlike systematic reviews or meta-analyses, methodological quality did not limit search criteria. Although attempts were made to report significant outcomes, our criteria may have underrepresented the absolute frequency of nonsignificant findings reported.

Most studies only included adults with quiescent or mild CD, excluding those with active or moderate–severe CD, and most intervention trials were of short duration, report small sample sizes and poor retention, and lack objective measurements of disease activity. The lack of adequately powered RCTs in this area might be because of challenges associated with securing extramural funding, likely due to lack of awareness and understanding about the level of disability of CD and the negative stigma oftentimes associated with diseases impacting the bowels.^9^ Nonetheless, performing pilot research is important for reducing type II errors and determining acceptability and potential benefits and harms of an intervention,^56^ and should lead to the eventual publication of larger, more stringent RCTs that allow for better evaluations of outcomes of PA in persons with CD.

One reason for the poor retention in the interventions included in this scoping review may be due to the lack of focus on PA promotion or adherence to PA. Indeed, the interventions included in this scoping review were based on formal exercise training programs, providing equipment to participants and teaching routines for exercise. These exercise programs are difficult to maintain since they usually require purchasing equipment or memberships after the intervention ends. By contrast, theory-based interventions that combine exercise training with cognitive and behavior change strategies are more effective at increasing and maintaining PA.^57^ This multicomponent approach recognizes that that PA cannot be influenced directly; instead, whether a person chooses to be physically active is influenced by a complex, interrelated set of mediators of PA behaviors, including psychosocial and environmental factors.^57^ Unfortunately, despite an extensive evidence base demonstrating the efficacy of interventions based on theory in promoting sustained participation in PA in similar populations,^58^ none of the interventions included in this scoping review mentioned the use of behavior change strategies to promote PA and increase adherence. To the best of our knowledge, the psychosocial and environmental factors that influence PA behaviors in adults with CD have not been studied, making this a vital area for future research.

## Conclusion

This scoping review synthesized existing evidence regarding PA rates, determinants, health consequences, and interventions into a single resource that can be used to inform future research efforts. Current self-report and objective measures of PA rates vary widely, but adults with CD appear to be similar to slightly less PA than the general population. PA may be associated with a reduced risk of future active disease in CD patients in clinical remission, as well as improve HRQOL, fatigue, and depression. Though the benefits of structured PA interventions on CD symptoms warrants further investigation, preliminary findings demonstrate that moderate-intensity PA is feasible, safe, and may have beneficial effects on disease activity. Overall, the benefits that can be accrued from regular PA are quite diverse; however, a substantially larger body of evidence is needed to provide firmer conclusions on the health benefits of PA that might underlie exercise-related changes in function and disease activity in adults with CD. Specifically, the results of this scoping review underscore the need for future studies to: (1) examine the psychosocial and environmental parameters that influence PA; (2) develop innovative, large-scale theory-based interventions that combine cognitive and behavioral change strategies for increasing and maintaining PA; and, (3) incorporate effective interventions that are inclusive of adults with moderate–severe CD. This can allow for better contextualization of the role of PA on the clinical course of CD as well as extraintestinal manifestations associated with the disease.

## Data Availability

No new data were created or analyzed.
